# Implantation of a biodegradable rectum balloon implant: Tips, Tricks and Pitfalls

**DOI:** 10.1590/S1677-5538.IBJU.2016.0494

**Published:** 2017

**Authors:** Ben G. L. Vanneste, Kees van De Beek, Ludy Lutgens, Philippe Lambin

**Affiliations:** 1 Department of Radiation Oncology (MAASTRO), GROW - School for Oncology and Developmental Biology, Maastricht University Medical Center+, Maastricht, Netherlands; 2 Department of Urology, Maastricht University Medical Center+, Maastricht, Netherlands

**Keywords:** Prostatic Neoplasms, Radiotherapy, Biodegradable Plastics

## Abstract

**Introduction:**

A rectum balloon implant (RBI) is a new device to spare rectal structures during prostate cancer radiotherapy. The theoretical advantages of a RBI are to reduce the high radiation dose to the anterior rectum wall, the possibility of a post-implant correction, and their predetermined shape with consequent predictable position.

**Objective:**

To describe, step-by-step, our mini-invasive technique for hands-free transperineal implantation of a RBI before start of radiotherapy treatment.

**Materials and Methods:**

We provide step-by-step instructions for optimization of the transperineal implantation procedure performed by urologists and/or radiation oncologists experienced with prostate brachytherapy and the use of the real-time bi-plane transrectal ultrasonography (TRUS) probe. A RBI was performed in 15 patients with localised prostate cancer. Perioperative side-effects were reported.

**Results:**

We provide ‘tips and tricks’ for optimizing the procedure and proper positioning of the RBI. Please watch the animation, see video in https://vimeo.com/205852376/789df4fae4.

The side-effects included mild discomfort to slight pain at the perineal region in 8 out of 15 patients. Seven patients (47%) had no complaints at all. Two patients developed redness of the skin, where prompt antibiotic regimen was started with no further sequelae. One patient revealed a temporary urine retention, which resolved in a few hours following conservative treatment. Further no perioperative complications occurred.

**Conclusion:**

This paper describes in detail the implantation procedure for an RBI. It is a feasible, safe and very well-tolerated procedure.

## INTRODUCTION

Prostate cancer radiotherapy can develop limiting anorectal toxicity (1–3). It is therefore important to implement techniques to spare rectal structures (3).

**Figure 1:**
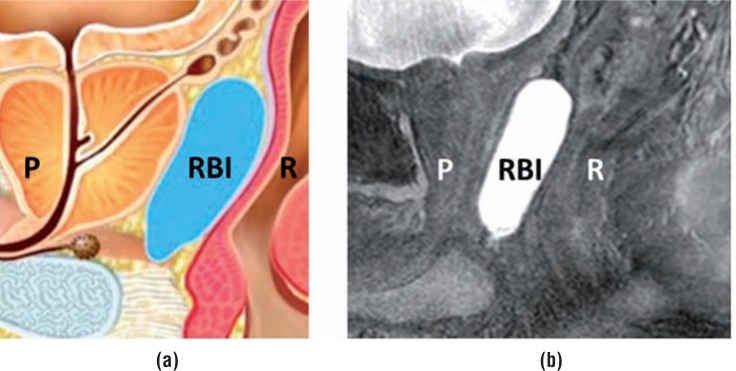
A schematic illustration (www.bioprotect.co.il) (a) and an MRI image (Balanced fast field Echo- sequence) (b) of a biodegradable rectum balloon implant (RBI) between the anterior rectum wall (R) and the prostate (p), creating space between the two organs.

**Figure 2:**
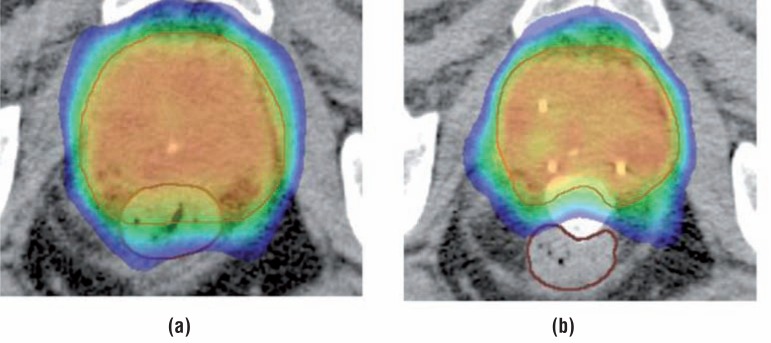
Isodose distribution in an axial cT plane before (a) and after rectum balloon implant (RBI) implantation (b) in the same patient. The image on the left shows the high-dose region >80% (green) overlapping the entire part of the rectum (brown), whereas with the RBI *in situ* the high-dose region is not in the rectum. The 65% isodose contour (blue) overlaps the entire rectum in (a), whereas there is no overlap in the rectum in (b).

Several devices have been established to spare anorectal structures by excluding them from high radiation dose exposure. Endo-rectal balloons are used to increase the distance from the dorsal rectal wall to the prostate (3), and implanted rectum spacers (IRS) are designed to separate the anterior rectal wall from the prostate by injecting a biodegradable material. Four types of IRS have been developed: hyaluronic acid (4), absorbable hydrogel (5), collagen implants (6), or a saline—filled balloon (7). In the past decade, research groups have investigated the use of a prostate IRS (Figure-1), with hyaluronic acid and poly-ethylene-glycol (PEG)-based hydrogel (4, 5, 8–12). All reported a decrease of the rectal dose (Figure-2).

**Figure 3:**
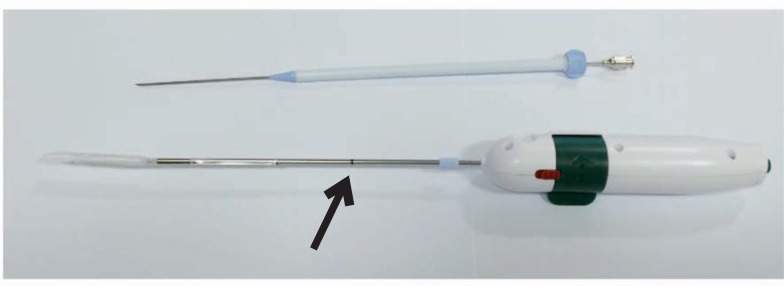
The RBI kit, with the needle with the dilatator (blue) and sheath (white) above, and the rectum balloon implant (rolled up) deployer (RBID) below. note the line on the RBID: when the RBID is inserted through the sheath up to this mark it means the tip of the deployer is at the end of the sheath. Retract the sheath while holding the RBID in place.

This paper describes in detail the implantation procedure for a (saline-filled) rectum balloon implant (RBI) (Figure-3). It provides step-by-step instructions, identifying the potential hazards and ‘tips and tricks’ for optimising the procedure as well as proper positioning of the RBI. Furthermore, we report the perioperative complications of the first 15 patients implanted in our institute.

## MATERIALS AND METHODS

After approval by the local ethics committee and institutional review board, 15 consecutive patients with localised prostate cancer (cT1-2 N0) treated between June 2015 and December 2015 were included in this feasibility study. Gleason scores > 7 and high PSA-values were not exclusion criteria. Extended extra-prostatic disease extension (T3a/4) was an exclusion criterion, as were distant metastatic disease and previous pelvic EBRT. All patients signed an informed consent document. The RBI (BioProtect Ltd, Israel) implantation was demonstrated in a video review to illustrate a clinically useful step-by-step technique see video in https://vimeo.com/205852376/789df4fae4. All patients were assessed immediately post-injection, 4 to 7 days after implantation. The possible complications were recorded in terms of Common Terminology Criteria for Adverse Events (Version 4.0) (13). Pain was scored 1 hour, 8 hours, and 24 hours after implantation using the visual analogue scale (VAS), ranging from 0 to 10.

## RESULTS

### Step-by-step description of application technique

#### Precautions - medications

Anticoagulation should be stopped before this minimally invasive procedure because bleeding can disturb transrectal ultrasound (TRUS) vision. The timing of therapy stop and re-initiation depends on the specific drug used. In contrast to transrectal biopsies, the transperineal RBI implantation yields a lower infection risk after careful skin preparation. Nevertheless, an antibiotic prophylaxis is recommended to reduce the risk of infection by the implant (12). A rectal enema will empty the rectum and improve the conditions for TRUS (14). We use oral ciprofl oxacine (500 mg, bid, for three days) and Colex Klysma (100mL, one hour before procedure).

#### Positioning - material

**Figure 4:**
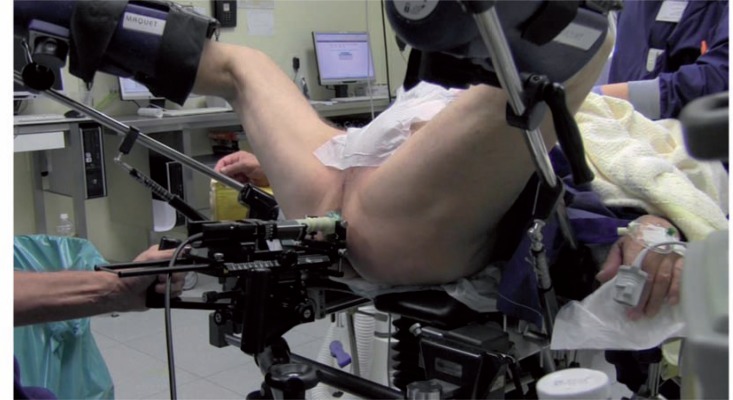
The setup: patient is placed in dorsal lithotomy position, with brachytherapy stepper unit and TRUS probe.

The RBI implantation is performed under TRUS guidance using the transperineal approach, with the patient placed in the dorsal lithotomy position (Figure-4). This setup is similar to the implantation procedure for prostate brachytherapy (9). A brachytherapy stepper unit is used to stabilise the TRUS probe and allows the operator to use both hands for the implantation.

A bi-plane TRUS probe (Pro Focus 2202 - BK Medical; transducer type 8848) is used with a US contrast gel-filled condom to improve visibility of the prostate, the Denonvilliers' fascia (DF) and the anterior rectal wall.

#### Anaesthesia

The implantation procedure can be performed under local, spinal or short general anaesthesia. A short general anaesthesia is preferred at the MAASTRO Clinic.

#### Procedure

First, a Foley balloon is inserted to empty the bladder so there is no resistance when the RBI is fully deployed, and to provide anatomical landmarks of the central plane, which consequently aids the central and effective positioning of the RBI.

Careful skin disinfection is performed with chlorhexidine solution (1%) 10mg/mL, and sterile drapes are used to cover the patient's legs.

Fiducial intraprostatic markers are implanted for image-guided external beam prostate irradiation. These gold markers could pierce and defl ate the RBI; to avoid this, we implant the fiducials via the transperineal (instead of transrectal) route simultaneous with the RBI implantations, as described by Gez et al. (15). We start implantation of the markers just before RBI implantation.

#### Hydrodissection

**Figure 5:**
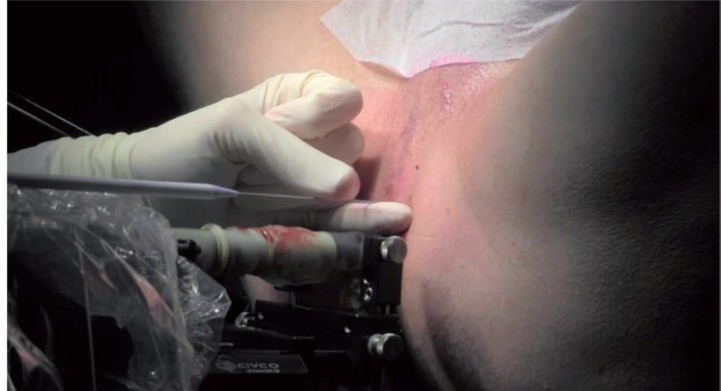
Transperineal insertion of the needle, with the dilatator (blue) and the sheath (white).

**Figure 6:**
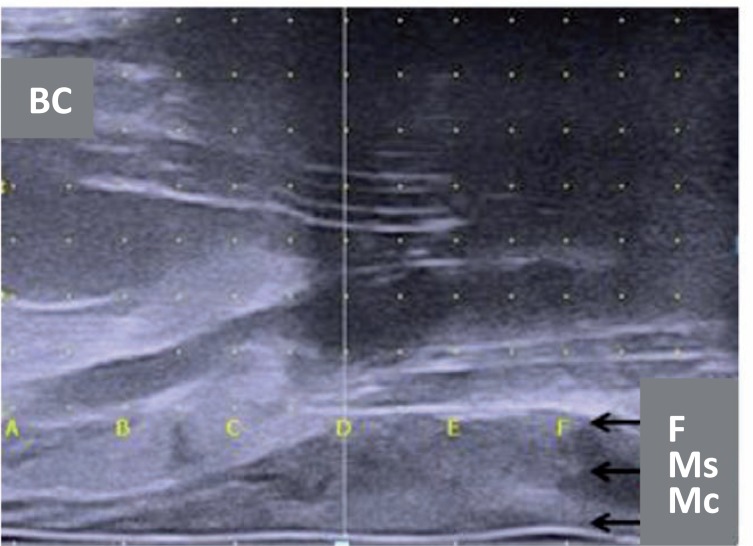
A hydrodissection is performed to separate the tissue planes with saline, helping to create space for the RBI between the Denonvilliers' fascia and the anterior rectal wall. Be mindful of the three layers of the rectum: fascia (f), muscle (Ms) and mucosa (Mc). The vertical white line is the base of the prostate. Most of the prostate is not clearly visible because of the acoustic shadow of the needle. note the foley balloon catheter (Bc) in the bladder and the catheter in the urethra, indicating that you are in the midline.

A hydrodissection using saline is performed to create tissue planes and facilitate correct placement of the RBI between the DF and the anterior rectal wall. A 20mL syringe is filled with saline. The needle is introduced through the perineum in the midline, ±1.5cm above the TRUS probe (little finger width) (Figure-5). This can easily be viewed on the axial TRUS view. Next, the needle must be introduced parallel to the probe (or slightly angled) into the prostate apex (switch to sagittal TRUS view). The hydrodissection is performed between the DF and the rectal fascia while advancing the needle within this space up to the prostate base. The DF is a fibromuscular structure, fused with the posterior prostate and seminal vesicles. Lowering the probe (dorsal) without pressure on the prostate (in contrast with brachytherapy procedure) before starting hydrodissection may help to open the space. The saline is injected slowly. As the space opens, the needle is advanced until it reaches mid-gland (4, 6, 7, 14). This manoeuvre must be monitored on axial and sagittal TRUS views. The three layers of the rectum (mucosa, muscle, fascia) must be visually inspected to ensure that no rectal fascia is caught by the needle (Figure-6).

#### Balloon insertion

**Figure 7:**
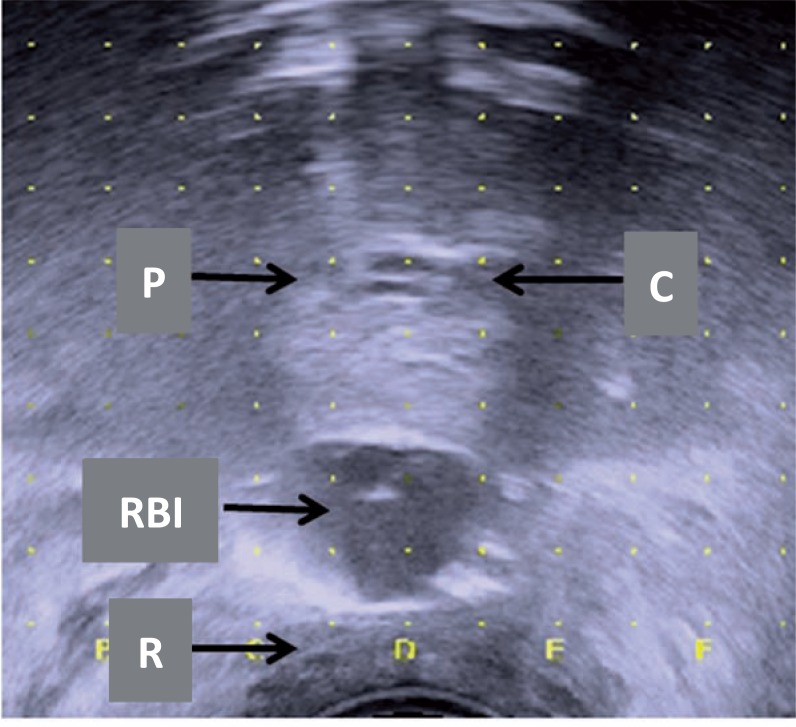
Axial ultrasound image: rectum balloon implant (RBI) being filled with saline between the prostate (p) and the rectum (R). note the urinary catheter (c) in the central plane, or ‘D-line’.

A 20mL syringe is filled with warm (35-40°C), bubble-free saline to fill the RBI. The saline is combined with 1.5cc contrast iodine to visualise the RBI on the planning CT and cone-beam CT scans. If the patient is allergic to contrast iodine, the RBI should be filled with saline only. The saline should be at body temperature to ensure adequate RBI expansion. Just superior of the needle, 1.5cm above the anus, a longitudinal skin incision of the perineum is made with 1 cm in width and 1.5cm deep. The dilatator is advanced with a sheath to the tip of the needle. Axial view is used to check that the dilatator and the sheath are in the central plane (‘D—line’, or plane of the urethral catheter). A switch to sagittal view is then made to advance the dilatator and sheath over the needle. The needle is shifted to check that the rectum wall is free. If it is not clear, a palpation is performed to check and feel if the rectum wall is free. When the sheath has advanced to the prostate base, the needle and the dilatator are removed while the sheath is firmly held in place. The RBI deployer (RBID) is inserted through the sheath up to the line marked on the RBID: the tip of the RBID will now be at the end of the sheath. The sheath is retracted while the RBID is held in place. The RBI is exposed and slowly inflated to the specified (15-20cc) volume, approximately 3mL every 3-5 seconds, while the inflation of the RBI is carefully checked on axial and sagittal views (Figures 6 and 7).

**Figure 8:**
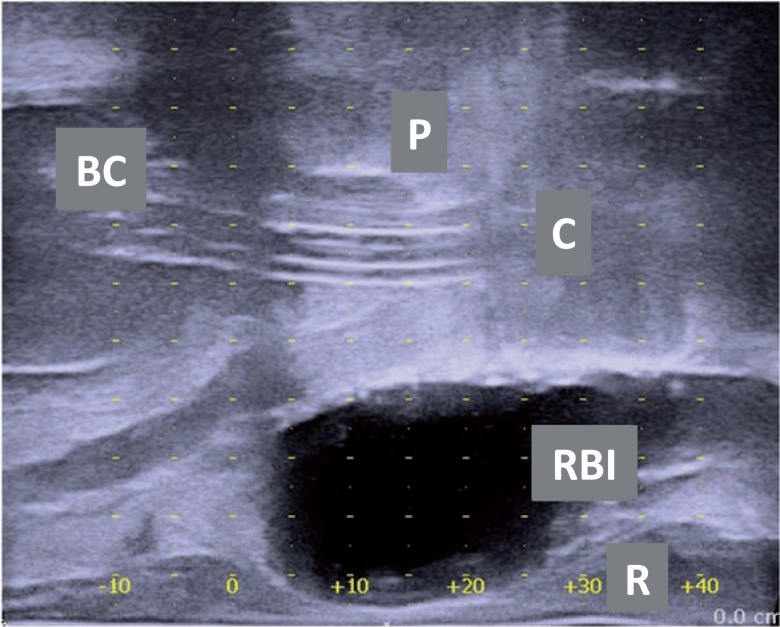
Sagittal ultrasound image of patient with a rectum balloon implant (RBI) *in situ* between the prostate (p) and the rectum (R). The view is in the central plane with the urinary catheter (C) and the foley balloon catheter (Bc) visible in the bladder.

Lowering the probe (dorsal) without placing pressure on the prostate may help to open the space and easily fill the RBI. The RBI must be in the midline between the prostate and rectum from base to apex. The three layers of the rectum (mucosa, muscle, fascia) must be visually inspected to ensure that no rectal fascia is caught by the needle (Figures 6 and 7). The TRUS probe is progressively moved down as far as possible, and a check is performed to verify that the rectum wall is free, in order to avoid rectum perforation. The RBID is detached from the RBI and left sealed *in situ* (7, 16). Axial and sagittal TRUS views are used to verify that the RBI is properly positioned (Figure-8). The rectal integrity and RBI position inflation are checked using rectal palpation. The skin incision is sutured using dissolvable stitches. Finally, the catheter is removed.

### Perioperative side-effects

No grade 3 or 4 toxicities were reported in the week after implantation. The implantation procedure revealed no thrombosis and no perforation of bladder or rectum, and no anti-allergic shock reaction occurred. No penile bleeding was observed. One patient experienced a temporary urine retention, which resolved within a few hours following conservative treatment.

There was a slight increase of redness of the skin in two patients, where a prompt antibiotic regimen was started with no subsequent episodes of infection.

The major side effect included pain in the perineal region (range from 1-3, according to VAS) in 5 out of 15 patients, which was easily addressed with paracetamol or nonsteroidal anti—inflammatory drugs. Three additional patients felt slight discomfort. Dysuria occurred in five patients. Ecchymosis in the transperineal region and tenesmus occurred in two patients and one patient, respectively. Seven patients (47%) were free of complications.

## DISCUSSION

The RBI separates the anterior rectal wall from the prostate, facilitating reduction of the high radiation dose to the anterior rectum wall. The potential failure modes, possible complications or pitfalls and corrective actions for this implantation procedure are described in Table-1.

**Table 1:** List of hazards adapted to RBI implantation and corrective actions.

Potential failure mode	Corrective action
**Bad TRUS view:**	
Stool	Rectal cleansing
Bubbles	Wait a few minutes
Prostate calcifications	Not reliable
**Hydrodissection:**	
**Needle is not advanced:**	
in the midline	Check relation on TRUS axial view and the D-line/central plane with the urinary catheter
to the prostate base	Palpate with finger to check if rectum wall is free
Not performed in the proper plane	Check on TRUS axial view and perform again
**Hydrodissection is not possible due to** incorrect position of the needle, e.g. in the rectum wall or in the prostate adhesions or patient anatomy	Reposition the needle Lowering the probe before starting may help to open the space; if this is not possible, it is recommended to abort the procedure
**Dilator:**	
is difficult to insert	Make a deeper incision
is not advanced to the prostate base	Check on TRUS and reposition
**Balloon:**	
cannot be inflated	Remove the sheath sufficiently
	Push the balloon deeper, so it does not interfere with pelvic muscles
is partially inflated and accidentally sealed	Remove RBI or detach it
is inflated in a suboptimal position (wrong cleavage)	Deflate RBI (percutaneous)
is sealed and spontaneously deflates	Completely Deflate RBI, be mindful of perforation
**Post-procedure:**	
Infection	Prophylactic antibiotic pre-procedure
	Quick start of antibiotic regimen
Bleeding	Stop antiplatelet therapy in advance
Urinary retention	Urinary catheter
Rectal perforation	Deflate RBI, suture, and post-operative antibiotics
Balloon is deflated	Implant transperineal fiducial markers before RBI implantation

Several types of spacers are available: hyaluronic acid, PEG-based hydrogel, human collagen, and biodegradable balloon. The advantage of the inflatable RBI system is that it allows for post—implant correction of the RBI position, whereas liquid spacers (hydrogels, hyaluronic acid, human collagen) do not permit any correction once injected (7). Furthermore, if such a liquid spacer is injected in the rectum wall, a rectum fistula can occur; this was recently mentioned by Habl et al., whereby they stopped using this promising technique (17). Next, the biodegradable RBI inflates to a predetermined and predictable shape, meaning a learning curve is probably less important. Pinkawa et al. reported a learning curve of 64 implantations to fully implement and optimise rectum hydrogel spacer placement (18). Therefore, we choose to use the RBI. However, a possible disadvantage is early volume loss of the RBI before the end of the radiation treatment, as recently published by Wolf et al. (19). Further research is needed to evaluate and quantify this volume loss.

The implantation of rectum spacers is well tolerated. No severe grade 3-4 complications occurred in our series. In the literature, severe complications have been documented, but in very low numbers (8, 9). Perforation of the bladder or rectum are reported in 3 out of 23 cases (13%) in procedures performed without hands-free TRUS guidance (5, 8). According to the authors, these complications resolved with no further sequelae. After protocol modifications and introduction of TRUS guidance, no perforations or other severe complications have occurred, as in our series. We observed 1 out of 15 cases (7%) of temporary urinary retention, probably provoked by the use of general anaesthesia. The literature reported this in 1 out of 11 cases (9%) and 3 out of 26 cases (12%), respectively (6, 15).

Most of the current literature is limited to spacer implantation in patients with low—risk localised (intra-capsular) prostate cancer. So far, the role of spacers in locally advanced and high-risk prostate cancers is unclear (8). The possible negative influence of a spacer in cases with a dorsal prostate capsule rupture is yet unknown, as tumour cells could be displaced out of the high-dose region by the spacer (14). Studies are therefore needed to evaluate the advantages and possible disadvantages of spacers in these patients.

Each RBI is handmade and has a variable maximum volume of 15-20cc (BioProtect Ltd, Israel). The volume must not exceed the specific amount indicated on the individual balloon label in order to preserve RBI function and prevent bursting (with consequent loss of function). In practice, we correlate the volume of the RBI with the volume of the prostate: small prostates (<35cc) do not need not the maximum RBI volume for sufficient space (at least 1cm). According to Pinkawa et al., a volume of 10mL is enough to ensure a distance of around 1cm (20).

Further clinical studies are required to define the place of an RBI in the treatment of prostate cancer radiotherapy. We believe that in the future, RBI should be prescribed on the basis of an individualised risk assessment with a validated predictive model and a decision support system to identify *a priori* whether individual patients will benefit from an RBI (21, 22). Prospective follow-up studies in independent patient cohorts are needed to assess the benefits of such an RBI.

## CONCLUSIONS

This paper provides detailed step-by-step instructions for the safe implantation of an RBI. This procedure should be performed by urologists and/or radiation oncologists who are experienced in prostate brachytherapy and the use of TRUS. The RBI implantation is a safe and very well tolerated procedure with only slightly increased discomfort, and in some cases pain in the perineal region, which is easily addressed with mild pain medication. The theoretical advantages of RBI include reducing the high radiation dose to the anterior rectum wall, the possibility of a post-implant correction, and the implant's predetermined shape with consequent predictable position, meaning a learning curve is probably less important.
